# Comparison of immunometabolic profiles in whole blood versus peripheral blood mononuclear cells

**DOI:** 10.1097/IN9.0000000000000073

**Published:** 2025-11-11

**Authors:** Ibrahima Diallo, Graham Heieis, Mikhael D. Manurung, Marouba Cisse, Yoanne D. Mouwenda, Yvonne C.M. Kruize, Moustapha Mbow, Maria Yazdanbakhsh, Bart Everts

**Affiliations:** 1 Center for Infectious Diseases, Leiden University Medical Center, Leiden, The Netherlands; 2 Department of Immunology, Faculty of Medicine, Pharmacy, and Odontology, Cheikh Anta Diop University of Dakar, Dakar, Senegal; 3 Centre de Recherches Médicales de Lambaréné, Lambaréné, Gabon

**Keywords:** metabolism, metabolic markers, immunometabolic profiling, whole blood, peripheral blood mononuclear cells, spectral flow cytometry

## Abstract

**Background:**

Immunometabolism has emerged as a flourishing field exploring how cellular metabolism regulates immune responses. Peripheral blood mononuclear cells (PBMCs) have so far been the primary sample type used for immunometabolic profiling. However, PBMCs isolation requires large blood volumes, can pose logistic challenges, and requires specialized skills for processing. Thus, using whole blood (WB) samples, which are less technically challenging to process, could serve as a viable alternative for metabolic characterization of circulating immune cell populations. Yet, how well WB immunometabolic profiles match those from PBMCs remains unknown. Therefore, we aimed to compare the immunometabolic profile of WB with that of PBMCs.

**Method:**

Paired WB and PBMCs samples were collected from six healthy donors. WB was collected in CryoStor^®^-CS10 medium, while PBMCs were isolated using Ficoll density gradient. Using spectral flow cytometry, we identified immune cell populations and assessed their metabolic states.

**Results:**

Our findings show an overall high similarity in the immune cell subset frequencies between WB and PBMCs as well as their metabolic profiles. However, differences in the expression of certain metabolic markers were noted in some immune populations. Specifically, glucose transporter 1 levels were higher in CD8^+^ TEMRA, NKT, and NK cells from PBMCs, while ATP5a levels were higher in naïve CD4^+^ T cells from WB.

**Conclusions:**

These results suggest that WB can be an alternative to PBMCs for metabolic profiling of immune cells. Nevertheless, for some specific cell subsets, caution should be taken when comparing immunometabolic data between WB and PBMCs.

## 1. Introduction

In recent years, immunometabolism has emerged as a flourishing field, contributing to a better understanding of how cellular metabolism impacts immune responses ^[1]^. The majority of studies on human immune-metabolic profiling have relied on the use of peripheral blood mononuclear cells (PBMCs), as they contain key immune cell types, including lymphocytes (including T cells and B cells) and innate immune cells such as NK cells, monocytes, and dendritic cells ^[2,3]^. However, the use of PBMCs has some drawbacks. PBMCs are not representative of all immune cell populations present in blood, as they lack granulocytes ^[4]^. Moreover, large volumes of blood, as well as specialized laboratory equipment and skilled personnel, are required for cell processing and storage ^[4]^, which are not always present in resource-limited settings. Therefore, investigations of immunological patterns using whole blood (WB) samples are increasingly gaining traction, in part due to recent improvements in storage methods for live WB cells ^[5,6]^, as well as the fact that WB is considered more physiologically relevant ^[7]^. Furthermore, for multicenter studies involving field work in remote areas in resource-poor settings, WB processing and storage may be more cost-effective than PBMCs isolation and cryopreservation.

Previous studies comparing fresh or cryopreserved WB with PBMCs suggested that the use of WB results in similar immune composition and functional outcomes to that of PBMCs ^[8]^. Importantly, for immunometabolic investigations, using WB over PBMCs might be advantageous, as it allows for the measurement of immunometabolic profiles of cells that have undergone fewer ex vivo processing steps ^[4]^ and therefore may provide a more accurate representation of the immune-metabolic state as it occurs in vivo. However, thus far, immunometabolic comparisons have not been performed between WB and PBMCs.

Until recently, studying immunometabolism required large cell numbers for bulk measurements and/or culture in media that led to global metabolic alterations not representative of the in vivo state. However, new single-cell approaches have been developed that help overcome cell number limitations. For instance, the use of fluorochrome (or metal) conjugated antibodies targeting metabolic enzymes or transporters for immunometabolic profiling by flow/mass cytometry has recently been validated ^[9,10]^. Thus, we took advantage of this approach to assess the metabolic properties of immune cells in small volumes of WB (200 µL), and to determine the similarities or differences of the profile in metabolic marker expression between WB and PBMCs across immune lineages. We report here that there is high concordance in immune composition between donor-matched PBMCs and WB samples. Regarding the metabolic profile, we found that the expression of six key enzymes or transporters (glucose transporter 1 [GLUT1], glucose-6-phosphate dehydrogenase [G6PD], succinate dehydrogenase complex subunit A [SDHA], ATP synthase F1 subunit alpha [ATP5a], CD98, and acetyl-CoA carboxylase 1 [ACC1]) showed global similarity in WB and PBMCs from the same donor. Despite the overall similarity, some noticeable differences were observed, particularly in GLUT1, which was restricted to effector lymphocyte populations. Additionally, ATP5a showed different expression patterns in CD4 naïve T cells. We therefore report that WB is a viable alternative to PBMCs for human immunometabolic studies. The results demonstrate the reliable use of WB in remote or resource-limited settings and provide a key reference for its use in multi-centric studies.

## 2. Materials and methods

### 2.1 PBMCs isolation and cryopreservation

For PBMCs isolation, 10 mL of blood collected in a sodium heparin tube (Cat: 455084; Greiner Bio-One VACUETTE, Kremsmünster, Austria) was transferred into a 50 mL tube containing 15 mL of 1.077 g/mL Ficoll (Cat: 979028619; Apotheek, LUMC-Pharmacy, Leiden, The Netherlands). After a 25-minute spin at room temperature at 450 *g* with no brake applied, the top plasma layer was removed using a 3 mL plastic pipette until 1 cm above the white ring, which was then carefully collected and transferred into a new sterile 50 mL tube. Cells were washed with 40 mL of Roswell Park Memorial Institute Medium (RPMI) 1640 (Cat:22400-089; Gibco/Life Technologies, Massachusetts, USA) for 10 minutes at 450 *g* at room temperature, then resuspended in 10 mL 10% Fetal Bovine Serum (FBS)/RPMI for cell counting and a second wash. Cell pellet was resuspended in the appropriate volume of 10% Dimethyl Sulfoxide (DMSO) (cat: 34943-1L-M, Sigma Aldrich; Israel)/FBS to reach 10.10⁶ cells/mL for freezing. Cryovials were transferred into a cold Mr. Frosty container (Nalgene Thermo Scientific, cat 5100-0001, Roschester, New York, USA) (18 tubes, 1.0–2.0 mL), a plastic container filled with isopropyl alcohol, which helps gradually lower the temperature of immune cells from room temperature to −80 °C (Thermo Scientific, cat 5100-0001, Rochester, New York, USA). The vials were kept overnight at −80 °C and then transferred the next day into liquid nitrogen for long-term cryopreservation.

### 2.2 Whole blood collection and cryopreservation

Regarding WB processing, from fresh blood, 5 mL was collected into an Ethylenediaminetetraacetic Acid (EDTA) tube, 200 µL were added to 1 mL of CryoStor^®^-CS10 (Stem-Cell/Technologies Cat: 07930, Washington, USA) in a cryotube (1.8 mL cryovials: NUNC 363401, Thermo Fishr Scientific, Massachusetts, USA). The cryovials were placed into cold Mr. Frosty, transferred to a 4 °C fridge for 10 minutes, and then transferred to −80 °C overnight. The cells were transferred into liquid nitrogen the next day. PBMCs and WB cryopreservation were stored in liquid nitrogen for 1 month.

### 2.3 Ethics statement

Paired WB and PBMCs samples were collected from six healthy donors. Ethical approval for the study was obtained from the Senegalese Ethics Committee (# Protocol SEN20/56) on 21 August 2020. Written informed consent was obtained from each participant. All participants were healthy donors. This research was in line with the Declaration of Helsinki.

### 2.4 Cell staining

Cells (PBMCs and WB) were first thawed in a 37 °C water bath and transferred to a 15 mL tube containing 5 mL of prewarmed (37 °C) thawing media (20% Fetal Calf Serum (FCS) (Lot. no. 1886, Bodinco BV, Alkmaar, The Netherlands) RPMI) and washed for 5 minutes at 450 *g*. For the WB cells, 3 mL RBC lysis was performed on Ammonium Chloride Potassium lysis buffer made in-house (0.15 M NH_4_Cl (Merck 101145, Germany), 1 mM KHCO_3_ (Merck 104854, Germany), 0.1 mM EDTA (Invitrogen 15575-038, California, USA; protocol number: LUMC-M50)) and incubated for 2 minutes. Cells were washed a second time with 5 mL media (20% FCS RPMI), resuspended in 1 mL Phosphate-Buffered Saline (PBS) (Fresenius Kabi, M090001, The Netherlands), and distributed into 96 well conical (V) bottom plate.

For staining with metabolic markers, both WB and PBMCs cells were first labeled with a live/dead marker (Live/Dead Fixable Blue, Cat: L23105, Invitrogen/Thermo Fisher Scientific, California, USA) diluted 1:500 in PBS containing 1:20 True-Stain Monocyte Blocker (Cat: 426102, Biolegend, California, USA) and 1:50 FC block (cat: 14-9161-73, Invitrogen/Thermo Fisher Scientific, California, USA) and incubated for 15 minutes at room temperature in the dark. After incubation, the plate was washed with 180 µL PBS for 5 minutes at 450 *g*, followed by a second wash with 100 µL of Fluorescence-activated Cell Sorting (FACS) buffer. Samples from the same donor were barcoded with either CD45-AF700 (clone:2D1, company: Biolegend, California, USA) or CD45-BV605 (clone: HI30, company: BD Biosciences, New Jersey, USA), then incubated for 30 minutes in the fridge (4 °C) in the dark. The stained WB cells and PBMCs were washed twice with 100 µL of FACS buffer for 5 minutes at 450 *g* and then mixed for the same donor before two-step staining with extra and intracellular panels prepared in FACS buffer (2mM EDTA, 0.5% BSA (Bovine Serum Albumin; cat: 10.735.086.001, Roche, Mannheim, Germany) in PBS) (Table 1). The fluorochromes included in the panels were selected according to the Aurora Five lasers 2, and each was associated with its corresponding marker. To prevent dye interactions that could cause staining artifacts, Brilliant Buffer (Cat: 566385, BD Horizon Brilliant™ dyes, BD Biosciences, New Jersey, USA) was added to the antibody mix at a 1:10 dilution.

**Table 1:** Immunometabolic panel for spectral flow cytometry.

Markers	Fluorochromes	Dilution	Staining	Company	Catolog#	Clone	Location (headquarter)	country
CD27	SB645	1/20	Extracellular	Thermo Fisher	N.A	323	Waltham, Massachusetts	USA
CD4	SB550	1/50	Extracellular	Biolegend	N.A	SK3	San Diego, California	USA
CD56	BUV737	1/50	Extracellular	BD Biosciences	N.A	NCAM16-2	Franklin Lakes, New Jersey	USA
CD40	BV480	1/100	Extracellular	BD Biosciences	N.A	5C3	Franklin Lakes, New Jersey	USA
CD197	PE-Fire810	1/100	Extracellular	Biolegend	N.A	G043H7	San Diego, California	USA
HLADR	APC Fire810	1/100	Extracellular	Biolegend	N.A	L243	San Diego, California	USA
CD14	BV570	1/100	Extracellular	Biolegend	N.A	MSE2	San Diego, California	USA
CD45RA	V450	1/100	Extracellular	BD Biosciences	N.A	HI100	Franklin Lakes, New Jersey	USA
IgD	PE-CF594	1/100	Extracellular	BD Biosciences	N.A	IA6-2	Franklin Lakes, New Jersey	USA
CD19	BV750	1/200	Extracellular	Biolegend	N.A	HIB19	San Diego, California	USA
CD3	CD496	1/200	Extracellular	BD Bioscience	N.A	UCHt1	Franklin Lakes, New Jersey	USA
CD98	BUV395(hu)	1/200	Extracellular	BD Biosciences	3297412	UM7F8	Franklin Lakes, New Jersey	USA
IgM	PerCP efluor710	1/400	Extracellular	Thermo Fisher	N.A	SA-DA4	Waltham, Massachusetts	USA
CD8	BUV805	1/400	Extracellular	BD Biosciences	N.A	SK1	Franklin Lakes, New Jersey	USA
CD45	BV605	1/400	Extracellular	BD Biosciences	N.A	HI30	Franklin Lakes, New Jersey	USA
CD45	AF700	1/400	Extracellular	Biolegend	N.A	2D1	San Diego, California	USA
CD16	BUV563	1/800	Extracellular	BD Biosciences	N.A	3G8	Franklin Lakes, New Jersey	USA
ATP5a	DL488	1/200	Intracellular	Abcam	ab231692(ab236553)	N.A	Cambridge Biomedical Campus, Cambridge	United Kingdom
ACC1	PE/Cy7	1/1000	Intracellular	Abcam	ab272704(ab102903)	N.A	Cambridge Biomedical Campus, Cambridge	United Kingdom
G6PD	APC/Cy7	1/1000	Intracellular	Abcam	ab231828(ab102859)	N.A	Cambridge Biomedical Campus, Cambridge	United Kingdom
GLUT1	DL405	1/1000	Intracellular	Abcam	ab252403(ab201798)	N.A	Cambridge Biomedical Campus, Cambridge	United Kingdom
SDHA	AF647	1/2000	Intracellular	Abcam	ab240098(ab269823)	N.A	Cambridge Biomedical Campus, Cambridge	United Kingdom

ACC1, acetyl-CoA carboxylase 1; ATP5a, ATP synthase F1 subunit alpha; G6PD, glucose-6-phosphate dehydrogenase; GLUT1, glucose transporter 1; SDHA, succinate dehydrogenase complex subunit A.

For extracellular staining, 50 µL of the mixture was added to each well, and the plate was incubated for 30 minutes at 4 °C in the dark (in the fridge). After incubation, the cells were washed twice with 180 µL FACS buffer, then fixed using 100 µL of a 1:4 dilution of e-Bioscience Foxp3/Transcription Factor buffer (lot 2652794, BD Biosciences) and incubated for 1 hour at room temperature in the dark. Stained cells were washed with 180 µL of 1:10 BD Perm/Wash buffer (Thermo Fisher Scientific™, Invitrogen, cat. 00-8333-56) with distilled water.

For intracellular staining, a cocktail of metabolic markers was prepared in BD Perm/Wash buffer with brilliant buffer at 1:10. Fifty microliters of the mixture were added to each well and incubated for 2 hours at 4 °C in the dark (in the fridge). The cells were then washed twice with 150 µL and 200 µL of Perm buffer, respectively, and resuspended in 180 µL of FACS buffer (**Supplementary Figure S1,** https://links.lww.com/IN9/A3).

### 2.5 Flow cytometry

Cells were acquired using Cytek Aurora 5 lasers and 2 spectral flow cytometer (Cytek Biosciences, Amsterdam, The Netherlands). Before sample acquisition, the number of events to acquire was determined and the side scatter (SSC) and the forward scatter (FSC) settings adjusted using an unstained sample. Following the setting of parameters, QC samples, including unstained sample, each single-stained sample, as well as live/dead controls, were acquired. After acquisition on the study samples, data were unmixed using the QC samples and settings applied across all samples. Finally, the fluorescence of each fluorochrome was checked, and compensation was applied when needed before the data were exported for further analysis (**Supplementary Figure S1,** https://links.lww.com/IN9/A3).

### 2.6 Data analysis

For data analysis, WB and PBMCs samples from the same donor were debarcoded and manually gated using FlowJo version 10.9.0. Statistical analysis was performed using a paired *t* test, and visualization was conducted in R (version 4.4.3). OMIQ (omiq.ai) was used for unsupervised clustering and visualization.

## 3. Results

### 3.1 Comparison of cell subsets between WB and PBMCs

We first sought to determine differences in the immune cell composition in both samples from the same donor, including T cell subsets (CD4^+^, CD8^+^, and their naïve, central memory, effector, and NKT subsets), B cell subsets (naïve, switched memory, and unswitched), NK cell subsets (bright and dim), monocyte subsets (classical, intermediate, and nonclassical), and dendritic cells in both WB and PBMCs using a high-dimensional antibody panel for spectral flow cytometry, which comprised of antibodies against 16 lineage and activation markers and six metabolic enzymes. To minimize technical variation in staining, we used a barcoding approach: based on labeling with CD45 antibodies, to allow for pooled staining of donor-matched PBMCs and WB. (**Supplementary Figure S1**, https://links.lww.com/IN9/A3).

**Figure 1.**
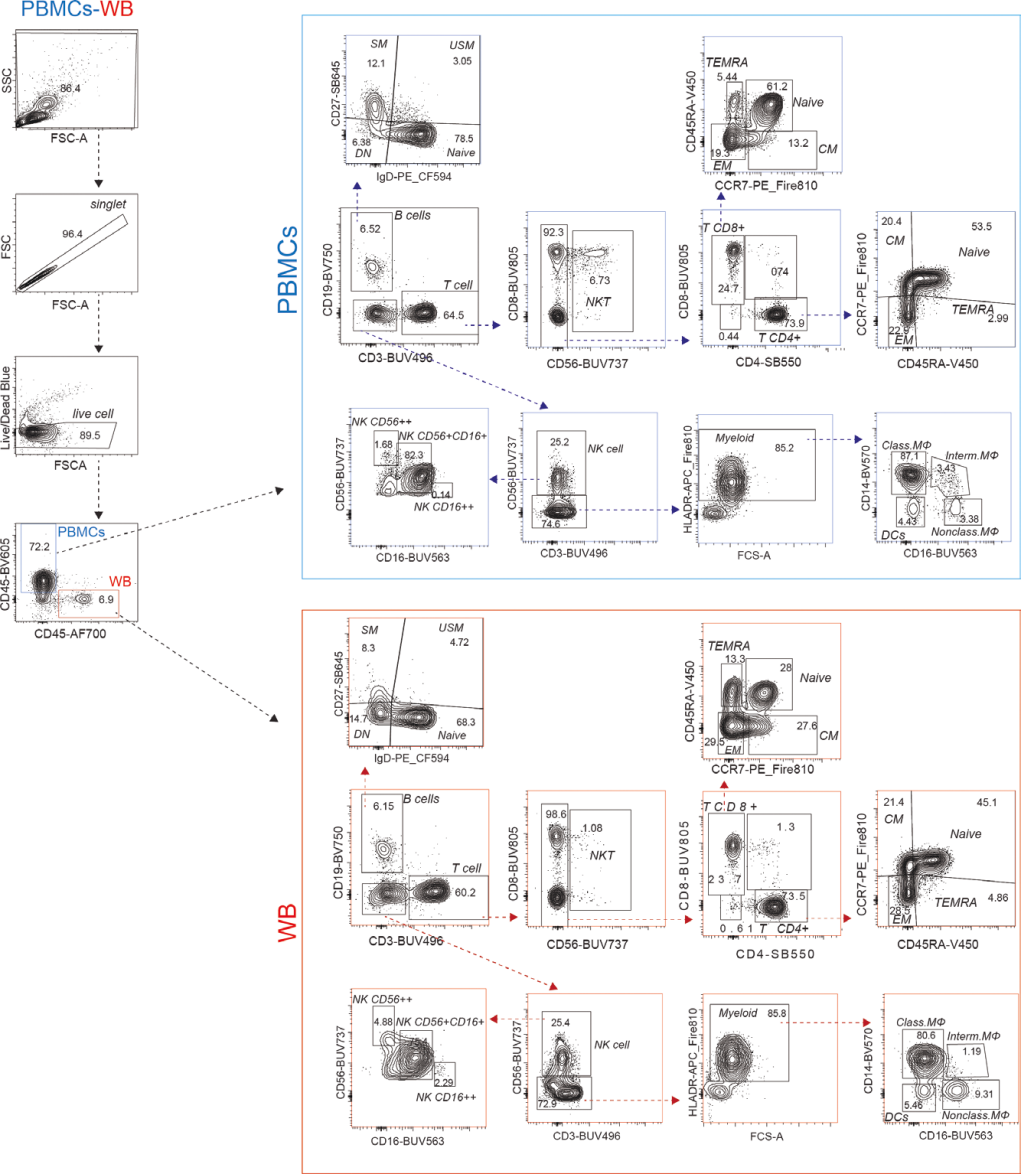
**Gating strategies in FlowJo to identify identical immune populations in WB and PBMCs.** Debarcoding CD45^+^ of WB and PBMCs for the same donor. Followed by manual gating strategies applied to identify T cell subsets: CD4^+^ T cells (central memory [CM], effector [EM], effector memory [EMRA], Naïve) and CD8^+^ T cells (central memory [CM], effector [EM], effector memory [EMRA], Naive); B cell subsets: (unswitched memory [USM], switched memory [SM], CD27^−^IgD^−^[DN], Naïve); NK cells (bright CD56 [CD56^+^CD16^−^], dim CD56 [CD56^−^CD16^+^] CD56^+^CD16^+^); myeloid cells (classical-, intermediate-, and non-classical monocytes as well as dendritic cells).

**Figure 2.**
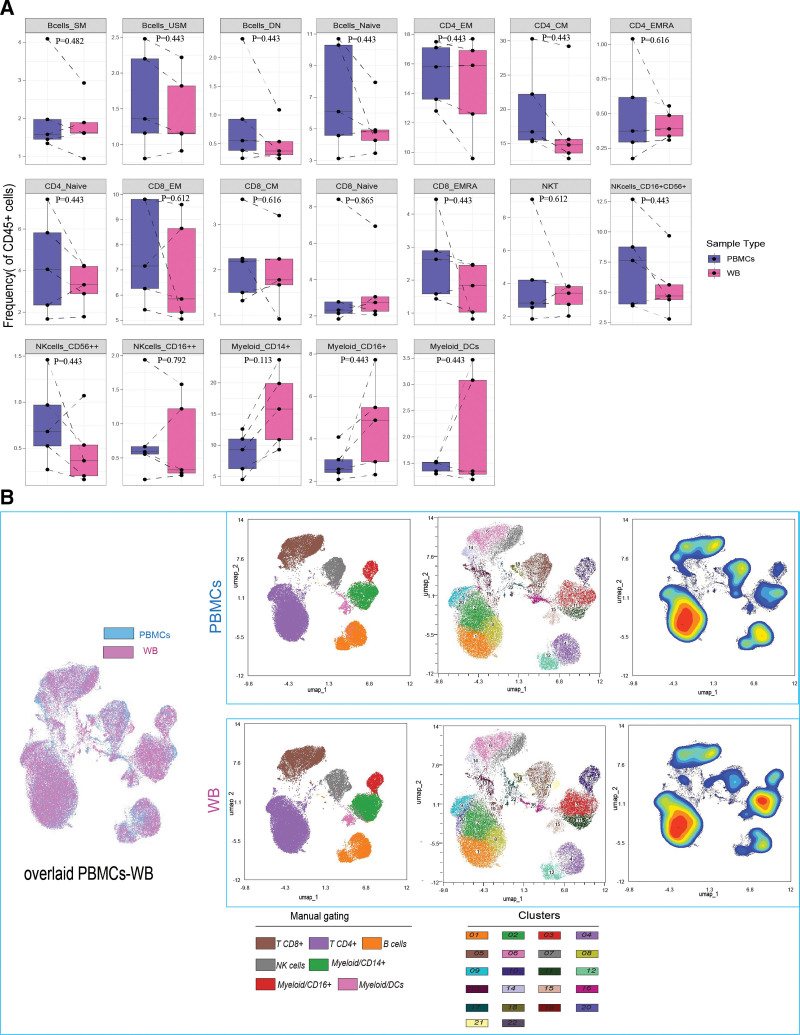
**Immunophenotyping profiles in WB and PBMCs.** (A) UMAP of PBMCs and WB overlaid after subsampling the same number of cells. UMAP shows a comparison of manual gating of major immune subsets and unsupervised clustering of PBMCs and WB from the same donor. Contour plots show the density distribution of cell populations. (B) Boxplots showing the frequency distribution of different immune cell subsets in WB and PBMCs. A paired *t* test was performed between WB and PBMCs, with multiple comparisons adjusted using the FDR correction (degrees of freedom, df = 4). FDR, false discovery rate; PBMCs, Peripheral blood mononuclear cells; UMAP, Uniform Manifold Approximation and Projection; WB, whole blood.

Comparative analysis of immune cell frequencies in WB and PBMCs samples was conducted using two approaches. First, manual gating was performed to define the main immune cell subsets (T cells, B cells, and myeloid cells) (Figure 1 and Table 2). Granulocytes, including neutrophils, were excluded from our analyses, as they are largely absent from PBMCs, which precludes their comparison between the two sample types (**Supplementary Figure S4**, https://links.lww.com/IN9/A6). The frequency distributions of the immune cell subsets were then compared between WB and PBMCs (Figure 2A). We did not observe significant differences in the frequency distribution of manually gated immune cell subsets of (Figure 2A). Secondly, unsupervised clustering with Phenograph was performed, which revealed 22 clusters. Here, unsupervised dimensionality reduction was visualized with Uniform Manifold Approximation and Projection (UMAP) using lineage markers. Cells were subsampled to ensure an equal number of cells from each sample type. This approach focused solely on immune cell types present in both sample types, allowing for an overlay of their spatial distributions (Figure 2B). Moreover, high-dimensional data visualization indicated close alignment in staining patterns, indicating consistent immunophenotyping results in the immune subset level between the two sample types (Figure 2B). The frequency distributions of these 22 clusters were compared between WB and PBMCs (Supplementary Figure S2A, https://links.lww.com/IN9/A4), and marker expression levels of each cluster per sample type are shown in the heatmap visualization (Supplementary Figure S2B, https://links.lww.com/IN9/A4). Consistent with the manual gating, we did not observe significant differences in the frequency distribution of 22 clusters nor major differences in marker expression levels in each of these clusters from WB versus PBMCs.

**Table 2:** Marker combinations for identifying immune cell subsets.

Markers expression	Subsets	Lineages
CD45^+^,CD19^−^,CD3^+^,CD56^−^,CD4^+^,CCR7^+^,CD45RA^−^	CM	TCD4^+^
CD45^+^,CD19^−^,CD3^+^,CD56^−^,CD4^+^,CCR7^+^,CD45RA^+^	Naïve	
CD45^+^,CD19^−^,CD3^+^,CD56^−^,CD4^+^,CCR7^−^,CD45RA^−^	EM	
CD45^+^,CD19^−^,CD3^+^,CD56^−^,CD4^+^,CCR7^−^,CD45RA^+^	TEMRA	
CD45^+^,CD19^−^,CD3^+^,CD56^−^,CD8^+^,CCR7^+^,CD45RA^−^	CM	TCD8^+^
CD45^+^,CD19^−^,CD3^+^,CD56^−^,CD8^+^,CCR7^+^,CD45RA^+^	Naïve	
CD45^+^,CD19^−^,CD3^+^,CD56^−^,CD8^+^,CCR7^−^,CD45RA^−^	EM	
CD45^+^,CD19^−^,CD3^+^,CD56^−^,CD8^+^,CCR7^−^,CD45RA^+^	TEMRA	
CD45^+^,CD3^−^,CD19^+^,CD27^−^,IgD^+^	Naïve	B cells
CD45^+^,CD3^−^,CD19^+^,CD27^+^,IgD^−^	SM	
CD45^+^,CD3^−^,CD19^+^,CD27^+^,IgD^+^	USM	
CD45^+^,CD3^−^,CD19^+^,CD27^−^,IgD^−^	DN	
CD45^+^,CD3^−^CD19^−^,CD56^+^CD16^+^	Nkcells	Nk cells
CD45^+^,CD3^−^CD19^−^,CD56^+^CD16^−^	NK-bright	
CD45^+^,CD3^−^CD19^−^,CD56^−^low,CD16^+^	NK-dim	
CD45^+^,CD3^−^,CD19^−^,CD56^−^,HLADR^+^,CD14^+^CD16^−^	Classical	Monocytes
CD45^+^,CD3^−^,CD19^−^,CD56^−^,HLADR^+^,CD14^+^CD16^+^	Intermediate	
CD45^+^,CD3^−^,CD19^−^,CD56^−^,HLADR^+^,CD14^−^CD16^+^	Non-classical	
CD45^+^,CD3^−^,CD19^−^,CD56^−^,HLADR^+^,CD14^−^CD16^−^	Dcs	DCs

### 3.2 Immunometabolic profile between whole blood and PBMCs

**Figure 3.**
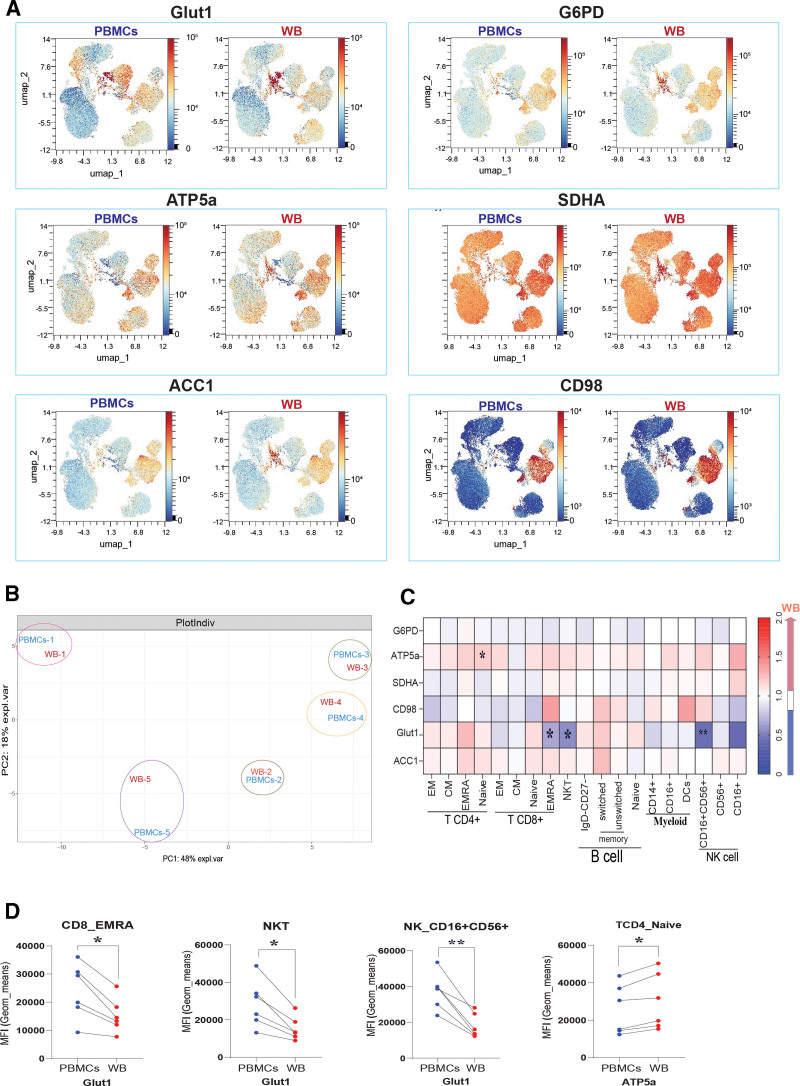
**Immunometabolic profiles of WB and PBMCs**. (A) UMAP, as shown in Figure 2B, in which expression levels of metabolic enzymes are shown (G6PD, ATP5a, SDHA, CD98, GLUT1, ACC1) in WB versus PBMCs. (B) PCA of WB and PBMCs from the same donor using the MFI of their metabolic enzyme markers. (C) Heatmap showing fold changes (WB/PBMCs) in enzyme expression across subsets with significant differences, determined by paired *t* test: GLUT1 (CD8^+^T cell EMRA [*], NKT [*], NK CD56^+^CD16^+^ [**]) and ATP5a (CD4^+^T cell naive [*]). (D) Parallel plots illustrating individual donor variation in enzyme expression for WB compared to PBMCs, where significant differences were observed (as shown in Figure 3C) by paired *t* test. (*) *P* < 0.5, (**) *P* < 0,01. ACC1, acetyl-CoA carboxylase 1; ATP5a, ATP synthase F1 subunit alpha; G6PD, glucose-6-phosphate dehydrogenase; GLUT1, glucose transporter 1; PBMCs, Peripheral blood mononuclear cells; SDHA, succinate dehydrogenase complex subunit A; UMAP, Uniform Manifold Approximation and Projection; WB, whole blood.

Next, we performed a comparative analysis of the cell metabolic phenotypes between paired WB samples and PBMCs, by analyzing the expression patterns of six metabolic enzymes or transporters by spectral flow cytometry that represent core metabolic pathways (glucose transport: GLUT1, amino-acid transport: CD98, pentose phosphate pathway: G6PD, fatty acid synthesis: ACC1, tricarboxylic acid [TCA] cycle: SDHA, and mitochondrial oxidative phosphorylation [Oxphos]: ATP5a). Of note, we stained for GLUT1 after cell permeabilization to allow for quantification of combined surface expression and of intracellular stores, as a proxy for glucose uptake potential. The expression of metabolic targets was visually compared using UMAP, which emphasized the heterogeneity of metabolic enzyme expression across immune subsets in both sample types, which between sample types displayed overlapping patterns (Figure 3A). For instance, GLUT1 expression was found to be highest in NK and B cells, as has been previously reported ^[11]^. Principal Component Analysis (PCA) plots were also generated based on metabolic target expression, revealing that WB and PBMCs from the same donors were largely similar, indicated by the high proximity of WB and PBMCs samples from the same donor relative to the same sample types from different donors (Figure 3B). However, at the immune subset level, discrepancies were observed in some subsets (Figure 3C). CD8^+^ TEMRA, NKT, and NK CD56^+^CD16^+^ cells showed higher expression levels of GLUT1 in PBMCs than WB. CD4^+^ T naïve cells showed the opposite result for ATP5a (Figure 3D**; Supplementary Figure S3A**, https://links.lww.com/IN9/A5). Furthermore, unsupervised clustering shows almost the same pattern compared with manual gating, revealing that metabolic differences were limited to five clusters **(Supplementary Figure S3B**, https://links.lww.com/IN9/A5), which largely correspond with differences observed for the manually gated immune cell populations. This suggests that, while immunometabolic profiles between WB and PBMCs are largely similar, there are subtle metabolic differences in specific immune cell subsets.

Overall, both WB and PBMCs show significant similarities in their immune composition and, despite some differences observed, metabolic phenotyping of WB and PBMCs reveals largely comparable trends.

## 4. Discussion

In this study, we asked whether WB and PBMCs display comparable metabolic phenotypes in immune cells. Overall, our data suggest that WB yields highly comparable results to PBMCs, which is largely consistent with previous immune phenotyping and functional studies in which PBMCs and WB were compared ^[3,4,7,12]^. This may further justify the recent increased interest of using WB for immunological studies ^[4,7]^.

While others have reported differences in B cell frequencies between WB and PBMCs ^[8]^, we did not observe this. These discrepant results could potentially be attributed to differences in freezing procedure, including a difference in the ratio of WB: CryoStor®-CS10 medium during freezing. A tendency for a decrease in monocyte frequencies in PBMCs compared with WB, we observed, is consistent with some loss of that cell population from the Ficoll isolation process, which has been previously reported ^[13]^. Despite the advantages of using WB, it should be noted that the restricted volumes of blood that can be stored in CryoStor^®^-CS10 freezing medium present some limitations. For instance, rare cells, such as innate lymphoid cells, may be missed due to their low frequencies ^[14]^. On the other hand, a major advantage of WB analysis is that it includes immune cell populations, such as granulocytes, that are absent from PBMCs. Due to their absence from PBMCs, we did not include them in our comparative immunometabolic analysis between WB and PBMCs. Of note, we found that red blood cell lysis, a crucial preparatory step for met-flow staining of the white blood cells in WB, did reduce granulocyte viability to some degree. Nonetheless, they could still readily be identified in the WB samples. This suggests that these cells can be subjected to met-flow analysis as well. Hence, the application of a met-flow panel with additional appropriate granulocyte markers would make it possible to comprehensively metabolically phenotype the whole circulating immune cell compartment.

Similar to the overall resemblance in immune cell composition, comparison of the metabolic phenotypes of the immune cells from paired WB and PBMCs samples also revealed largely overlapping metabolic profiles. Specifically, among 19 manually gated immune cell subsets, we identified only significant metabolic differences in four. Similarly, unsupervised clustering revealed significant metabolic differences in only five out of 22 clusters. Several processing steps that are different between WB and PBMCs could potentially contribute to this. First, density gradient separation used for PBMCs isolation has been reported to induce unwanted activation of immune cells ^[4]^, the latter of which is known to depend on increased glycolysis ^[15]^. Hence, higher expression in some PBMC immune cell subsets compared with WB of GLUT1, the main glucose transporter controlling glycolytic rates in immune cells, would be consistent with this ^[15]^. In addition, the lower ATP5a expression observed in naïve CD4^+^ T cells in PBMCs compared with WB may align with a shift towards dependence on glycolytic metabolism, at the expense of Oxphos. Second, immune cells exhibit rapid adaptability to in vitro culture conditions, which vary markedly from their native matrix (ie, blood) ^[16,17]^. Thus, a prolonged isolation at room temperature may be sufficient to drive metabolic adaptation of lymphocytes. Third, it cannot be ruled out that the use of two different anticoagulants, EDTA for WB cryopreservation and heparin for PBMC isolation, which are the standard anticoagulants used for these procedures, may contribute to the observed differences in immunometabolic profiles. For Instance, a study with porcine blood found that EDTA can reduce cytokine production in WB but enhances it in isolated PBMCs ^[18]^. However, to our knowledge, there are no studies that have linked EDTA or heparin usage to changes in immune cell metabolism, but this would be interesting to investigate. Finally, it is possible that red blood cell lysis, a crucial preparatory step for met-flow staining of white blood cells specifically in WB, may impact immunometabolic states. Further experiments would be needed to explore the contribution of the use of different anticoagulants and/or red blood cell lysis to some of the small differences in immunometabolic states we observed between WB and PBMCs.

Our work builds upon studies from Ahl et al ^[9]^ who introduced Met-Flow. These and other recent developments in the field of single-cell metabolic profiling now offer the chance to perform in-depth characterization of metabolic states and measurements of nutrient uptake at single-cell resolution ^[19,20]^, to study how immune cell metabolism adapts during activation, inflammation, and disease. This provides exciting new insights into the metabolic shifts that drive immune functions and immunopathology ^[21]^ in both infectious, inflammatory, and autoimmune diseases and cancer and may pave the way for exploring the potential of novel therapeutics that harness immunometabolism to treat these diseases ^[10,21]^. Our work suggests that for these types of investigations, WB could serve as a promising additional type of study material, as it preserves the full spectrum of metabolic activity across all blood components, including granulocytes, which may be underrepresented in PBMC preparations but play a critical role in inflammation and the body’s immune response ^[22,23]^.

Finally, we would like to point out that, although the currently applied met-flow is a very powerful single-cell metabolic analysis approach ^[9,24]^, it has its limitations, as it only provides a proxy for the activity of metabolic pathways that are covered by involved enzymes that are part of the met-flow panel. Application of additional metabolic profiling platforms, such as single-cell transcriptomics and metabolomics ^[25]^, would be needed to generate a more comprehensive and complete picture of the metabolic profiles of immune cells and their differences between WB and PBMCs. In addition, in the current experimental setup, we have only compared immunometabolic states ex vivo, without stimulation. Additional comparison of the metabolic profiles between PBMCs and WB following stimulation or activation would be helpful to map the immunometabolic similarities and differences across a wider range of conditions and to further strengthen our conclusions.

## 5. Conclusions

Overall, our study shows that WB can be a complementary source to PBMCs for immunometabolic profiling, with the added advantage of minimal ex vivo manipulations that could affect metabolic states, thereby potentially better preserving the in situ metabolic profile of immune cells. Altogether, our study provides a valuable reference for future investigation of the metabolic nature of immune cells using WB as source material.

## Conflicts of interest

The authors declare that they have no conflict of interest.

## Funding

This work was supported by grants from the Dutch Research Organization (NWO) through the Spinoza prize awarded to Maria Yazdanbakhsh and the European Research Council (ERC) via the ERC Advanced Grant “REVERSE” awarded to Maria Yazdanbakhsh (Grant No: 101055179).

## Acknowledgements

We thank all the participants who took part in this study. We also thank the Flow Cytometry Core Facility of LUMC.

## Supplementary Material


