# Supplementary figures and images for: Comparison of immunometabolic profiles in whole blood versus peripheral blood mononuclear cells
Source: Immunometabolism (Cobham). 2025 Nov 11;7(4):e00073. doi: 10.1097/IN9.0000000000000073 (PMC12604536; doi:10.1097/IN9.0000000000000073)

# Protocol

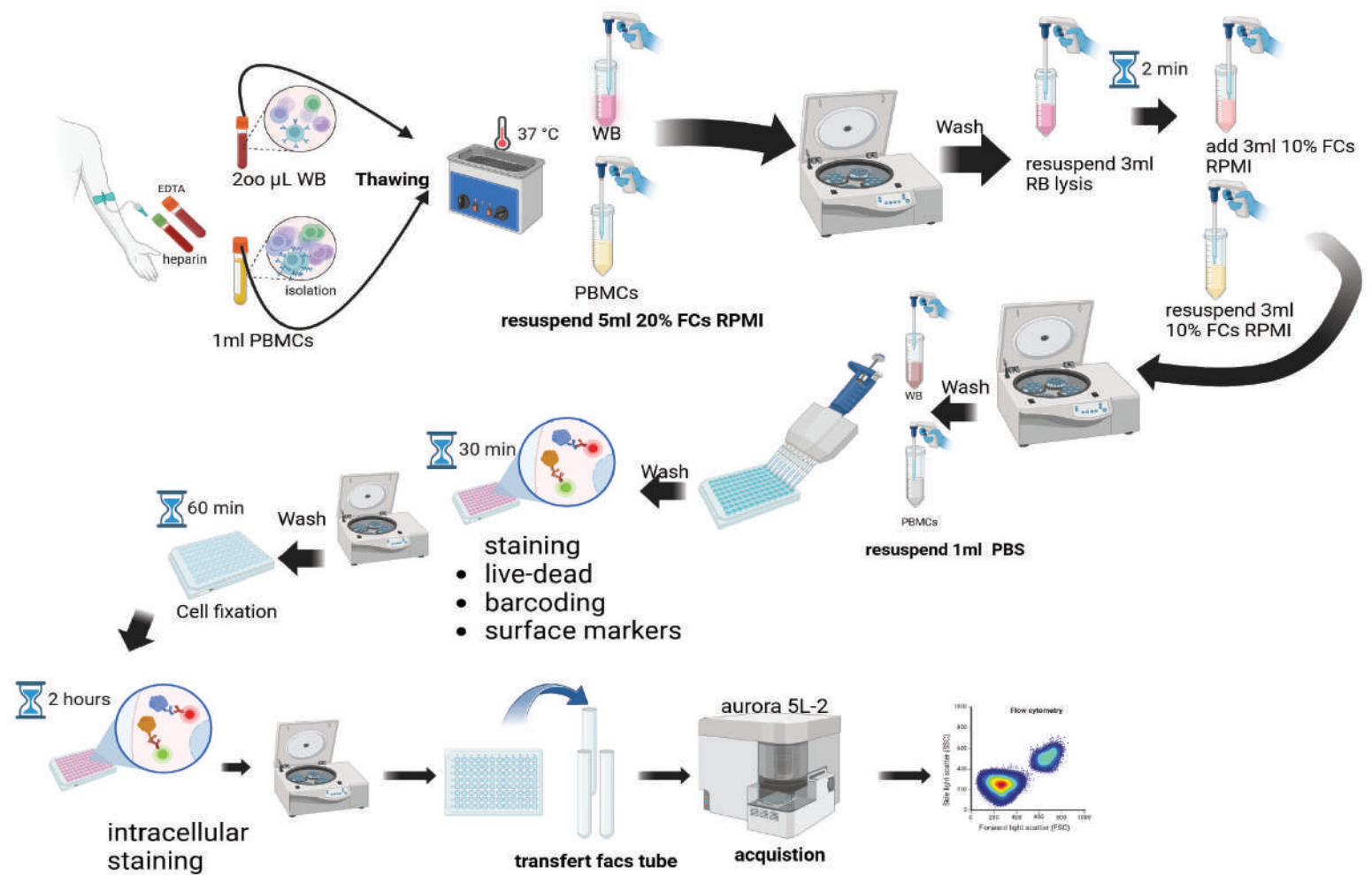

Supplement: Supplementary file 1 [file in9-7-e00073-s001.pdf]

A-

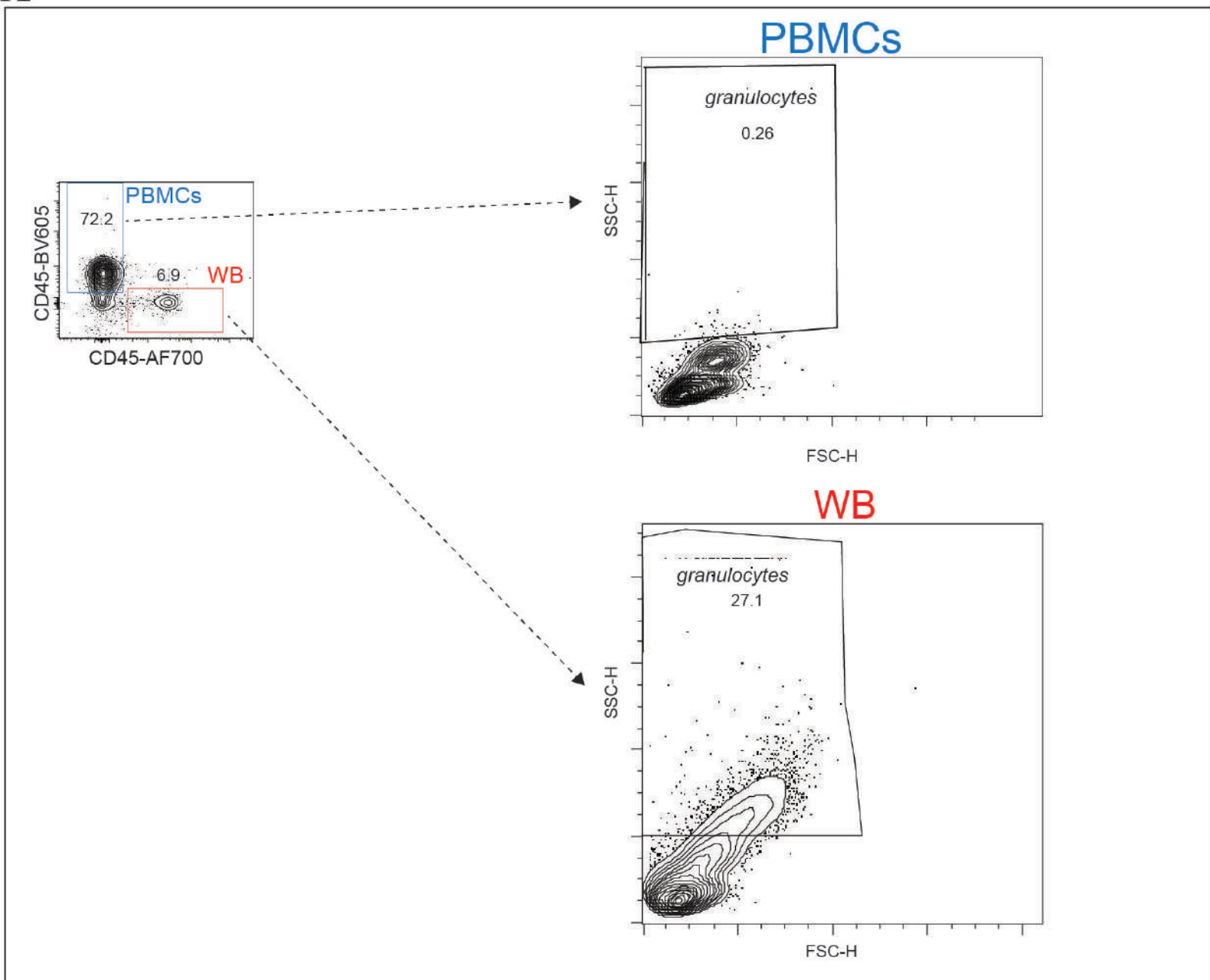

B-

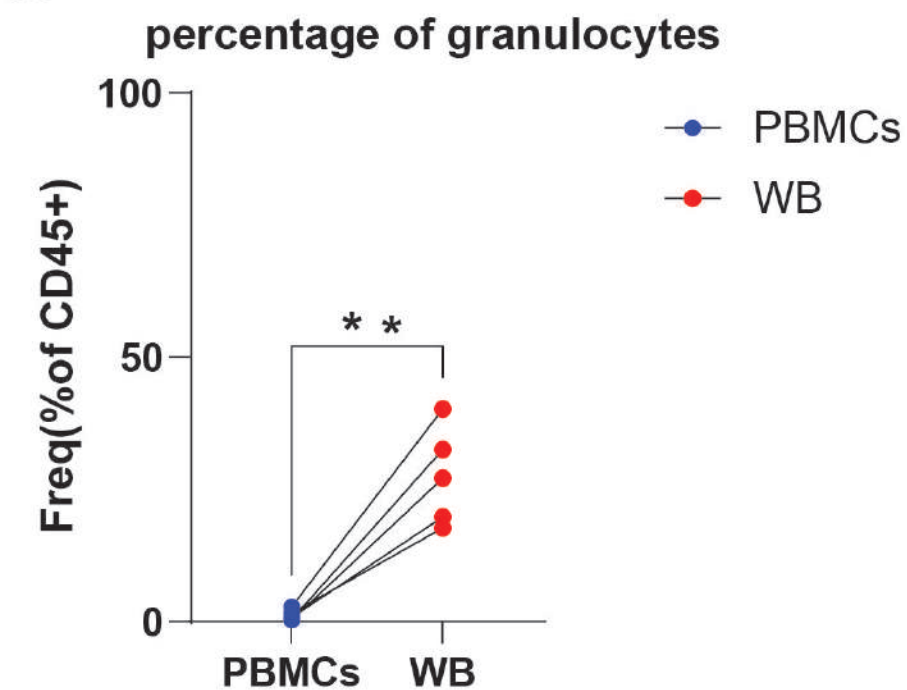

Supplement: Supplementary file 2 [file in9-7-e00073-s002.pdf]

A-

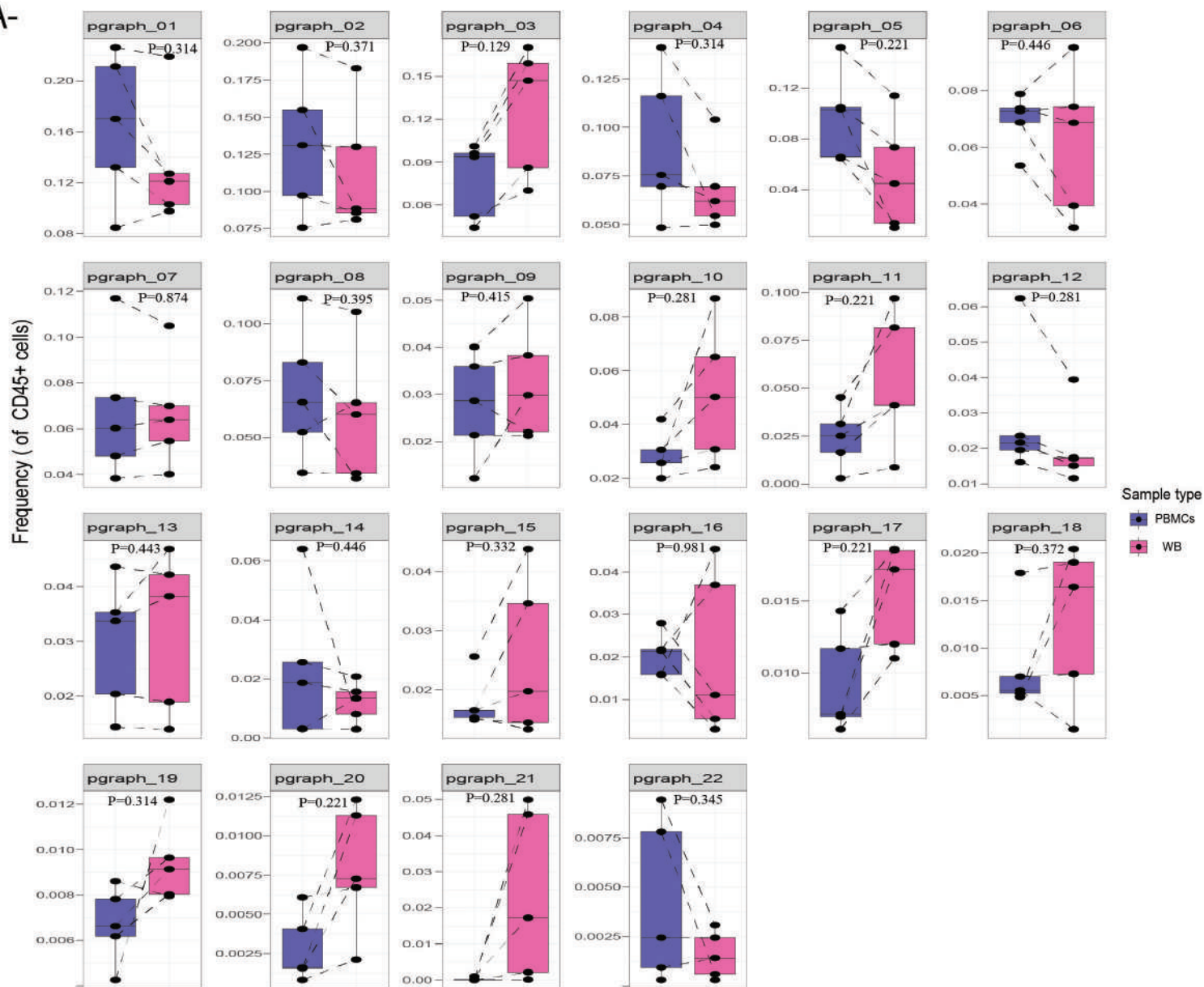

B-

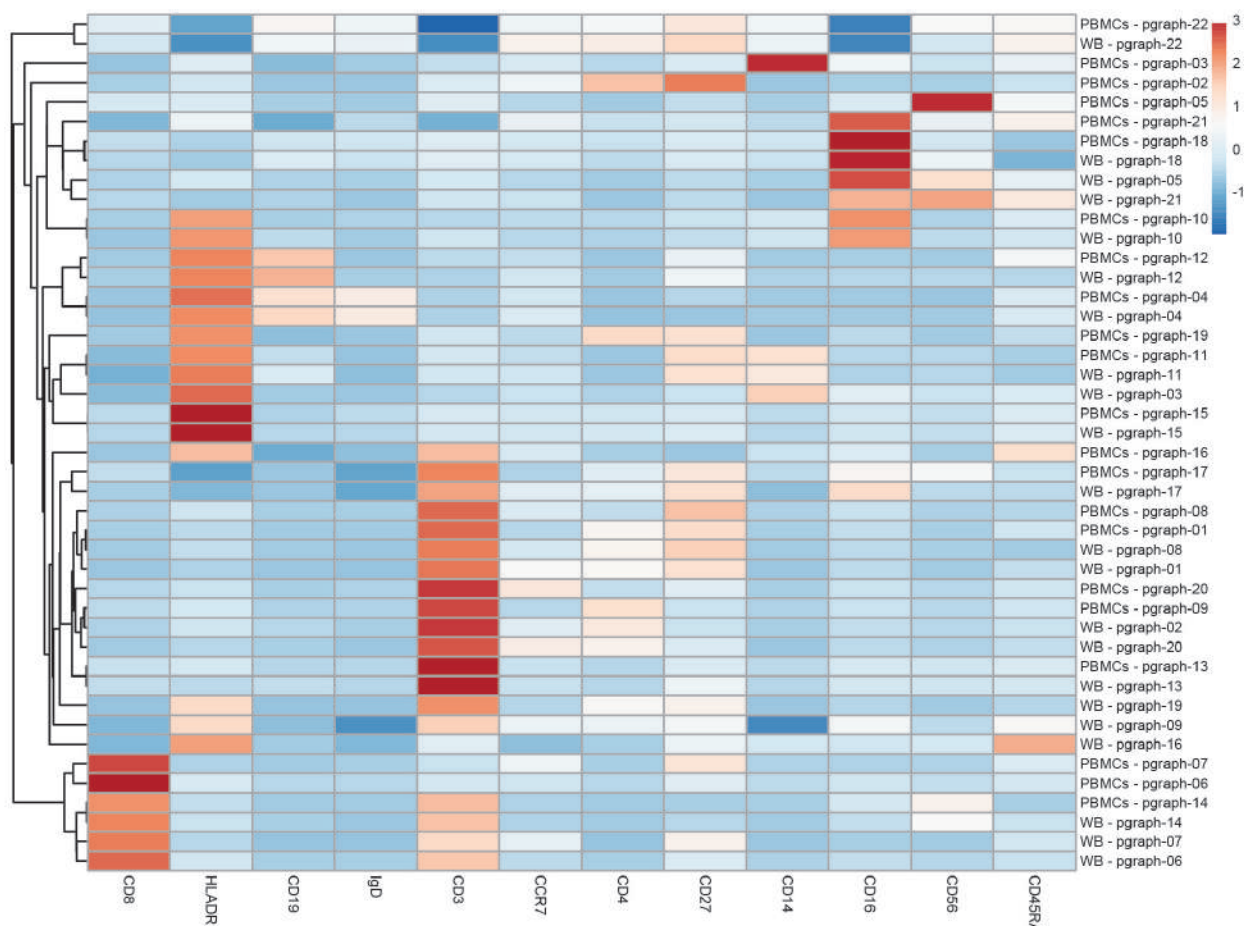

Supplement: Supplementary file 3 [file in9-7-e00073-s003.pdf]

A-

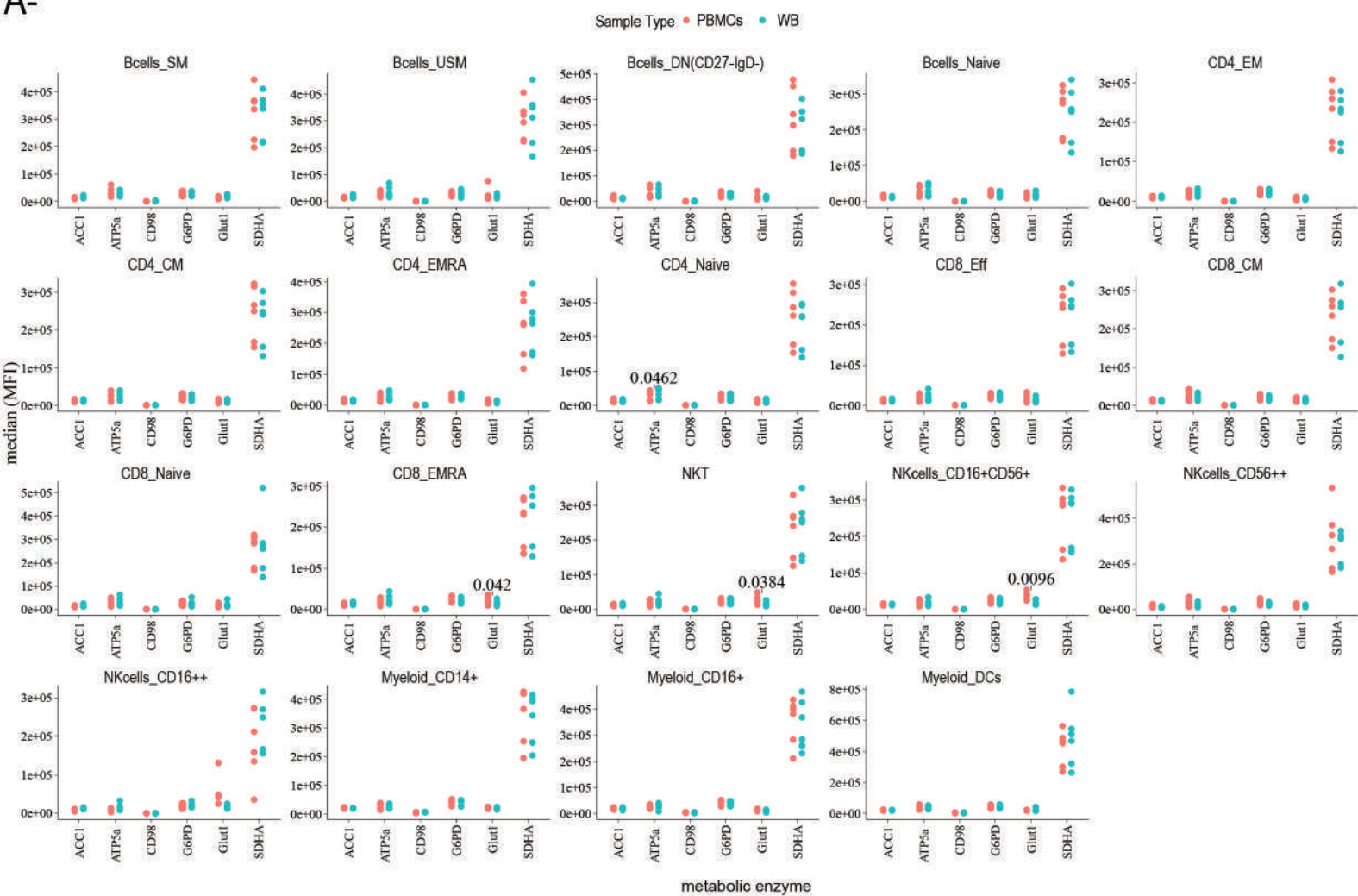

B-

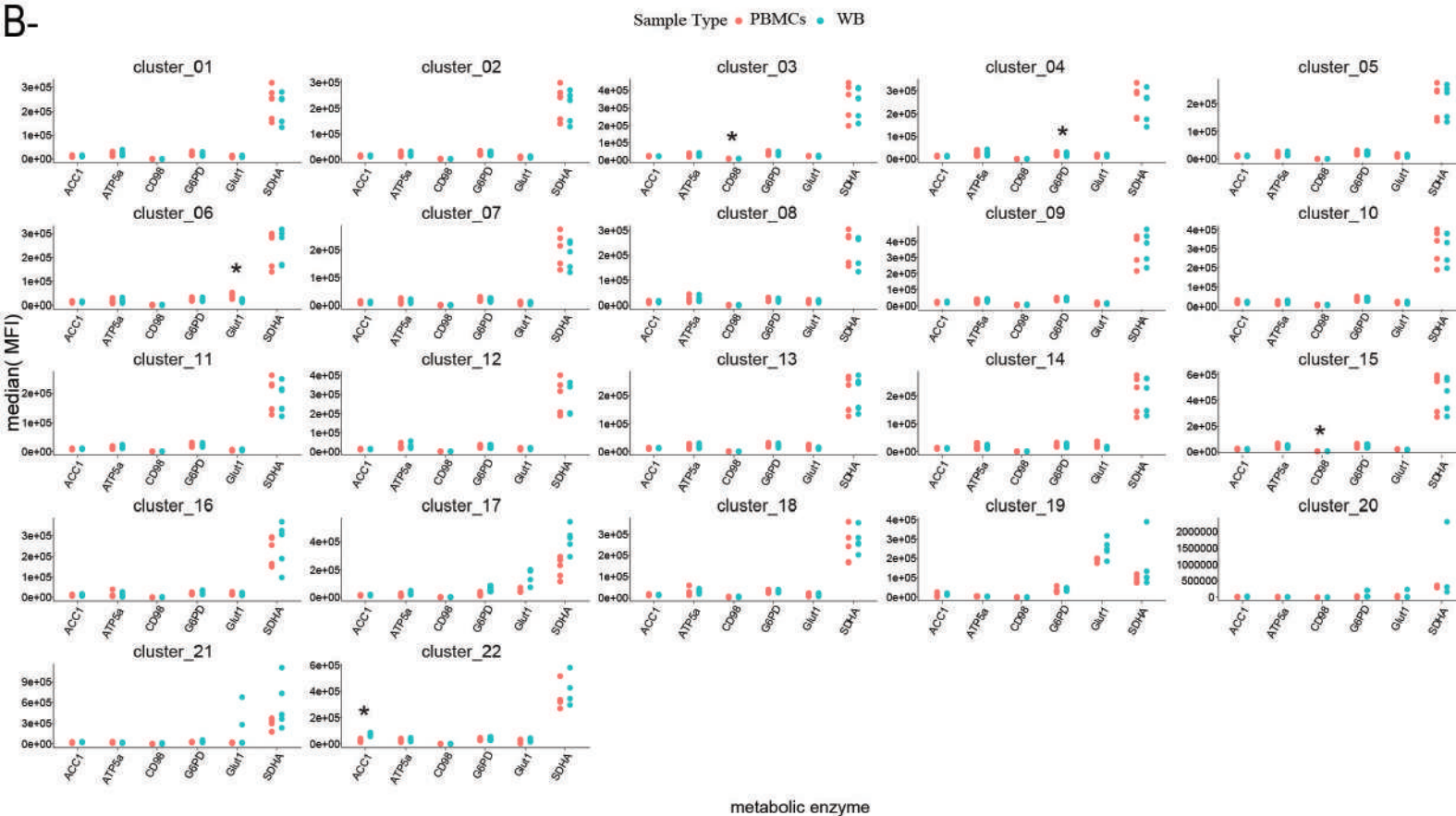

Supplement: Supplementary file 4 [file in9-7-e00073-s004.pdf]
